# Women’s Perceptions and Misperceptions of Male Circumcision: A Mixed Methods Study in Zambia

**DOI:** 10.1371/journal.pone.0149517

**Published:** 2016-03-03

**Authors:** Nicole A. Haberland, Christine A. Kelly, Drosin M. Mulenga, Barbara S. Mensch, Paul C. Hewett

**Affiliations:** 1 Population Council, New York, United States of America; 2 London School of Hygiene and Tropical Medicine, London, United Kingdom; 3 Population Council, Lusaka, Zambia; 4 Population Council, Washington DC, United States of America; Tulane University School of Public Health, UNITED STATES

## Abstract

Women’s perceptions of male circumcision (MC) have implications for behavioral risk compensation, demand, and the impact of MC programs on women’s health. This mixed methods study combines data from the first two rounds of a longitudinal study (n = 934) and in-depth interviews with a subsample of respondents (n = 45) between rounds. Most women correctly reported that MC reduces men’s risk of HIV (64% R1, 82% R2). However, 30% of women at R1, and significantly more (41%) at R2, incorrectly believed MC is fully protective for men against HIV. Women also greatly overestimated the protection MC offers against STIs. The proportion of women who believed MC reduces a woman’s HIV risk if she has sex with a man who is circumcised increased significantly (50% to 70%). Qualitative data elaborate women’s misperception regarding MC. Programs should address women’s informational needs and continue to emphasize that condoms remain critical, regardless of male partner’s circumcision status.

## Introduction

Randomized controlled trials have demonstrated that male circumcision (MC) reduces men’s risk of heterosexual acquisition of HIV [1–3]. Specifically, a circumcised man who has vaginal sex with an HIV-positive woman is about 60% less likely to contract HIV than a man who is not circumcised. The evidence that MC can reduce men’s risk of HIV infection led WHO in 2007 to recommend expansion of MC services in settings with generalized HIV epidemics and low prevalence of MC [4]. A number of countries in sub-Saharan Africa have initiated national voluntary medical MC programs, including Zambia [5, 6]. While pilot programs began performing VMMC’s in Zambia in 2008, the national program was officially launched in 2009 [6]. The number of VMMCs increased from fewer than 3,000 in 2008, to a cumulative total of approximately 82,000 by the end of 2010, to over 950,000 by the end of 2014 (WHO 2015).

These national efforts to scale up male circumcision raise the question of what impact MC may have on women. A randomized controlled trial in Rakai, Uganda found that circumcision of HIV-positive men had no significant effect on the likelihood of HIV transmission to uninfected female partners [7]. Notably, among couples that resumed sex early (i.e., more than 5 days before the male partner’s post-MC wound was certified as completely healed) women’s risk of infection was significantly higher at 6-months than among control group couples where the HIV-positive male was not circumcised. A systematic review and meta-analysis combining data from the Rakai trial with six longitudinal observational studies among sero-discordant couples similarly found no significant effect of MC on male to female transmission of HIV [8]. Subsequently, a prospective study of 1,096 HIV-sero-discordant couples in Africa also found no statistically significant effect of MC on women’s risk of HIV acquisition [9].

While studies to date among sero-discordant couples have not found any direct protective effect of MC from HIV infection for women, some level of reduced risk for women is expected via other pathways. One longer-term mechanism reducing risk is herd immunity, whereby benefits are expected for women at the aggregate level as the HIV prevalence declines in the male population [10]. At the individual level, MC may also offer protection in cases where, for example, a husband is HIV-negative but has extramarital partners. If the husband is circumcised, his risk of HIV infection is reduced (though not eliminated) and, by extension, his wife’s risk is reduced as well. Both these pathways, however, are indirect. Consequently, for an individual woman considering sex with a circumcised partner, his circumcision status is no guarantee that she will be free of risk of acquiring the virus.

Education regarding the effects of MC on HIV transmission must convey complex messages to both males and females. Accurate knowledge of MC entails understanding that MC is only partially protective against HIV for men, and that it does not provide direct protection against infection for women. Another source of possible confusion lies in the fact that MC reduces—but does not eliminate—the risk of acquiring some—but not all—STIs for men *and* for women [11–15].

While the multiple caveats regarding the protective effect of MC make for complex messaging, an accurate perception is important for several reasons. First, overestimating the efficacy of MC could result in risk compensation, whereby perceived protection leads men to reduce other protective behaviors such as condom use, monogamy, or age of sexual debut, and women to decrease their caution with respect to condom use or learning their partner’s HIV status. While evidence for risk compensation is limited within existing RCTs [16, 17], one prospective cohort study in Nyanza found no evidence of risk compensation in the context of Kenya's VMMC program [18].

Second, underestimating the protective effects of MC could undermine the demand for MC, both for adult males and for male children and infants. Westercamp and colleagues, for instance, found that the belief that MC reduces men's risk of HIV was significantly associated with preference for MC among both Kenyan women and men [19]. In the decision to circumcise male infants, one study found that when parents disagreed, the decision not to circumcise prevailed regardless of whether it was the mother or father who opposed MC [20].

Despite the critical importance of women understanding MC’s links with HIV infection and the limits of its protection, women’s perspectives have been largely overlooked, although there are some notable exceptions (for example, Westercamp *et al* [19]). Andersson and Cockcroft, using 2008 data from Botswana, Namibia and Swaziland, found that 10.2% of females and 13.0% of males believed that MC fully protects men from HIV, and that 18.5% of females and 17.6% of males believed that HIV-positive men who are circumcised cannot transmit HIV [21]. Another study in South Africa found that among a traditionally circumcising population, women who had heard that MC reduces a man’s risk of contracting HIV were significantly less likely to perceive themselves at risk of HIV infection, less likely to use condoms, and less likely to use condoms consistently with partners of positive or unknown HIV status, compared to women who had not heard this [22]. Similarly, a study among circumcised Kenyan males and their partners found that women were more likely than men to report attitudes that could lead to risk compensation [23].

This paper addresses an important gap in the literature by focusing explicitly on women’s perceptions of MC’s protective effects for both their partners and themselves. We combine quantitative and qualitative data from a population-based longitudinal study in Zambia to examine the breadth and depth of women’s awareness of the relationships between MC and HIV/STI transmission and the implications of this knowledge—or lack thereof—for MC uptake and post-procedure risk behavior in the context of a national VMMC roll-out.

## Methods

A mixed-methods approach was employed to explore the prevalence, depth, and correlates of women’s perception of MC’s protective effects [24, 25]. Specifically, we “nested” a data-linked qualitative subsample within a larger longitudinal survey to provide a more nuanced and complete understanding of MC-related knowledge [26].

### Household longitudinal survey

This paper uses data from the first two rounds of a prospective cohort study of post-MC sexual behavior in Zambia. The research sample comprised men and women aged 15–29 years at baseline, resident in seven of the nine provinces in Zambia. The Western and Northwestern provinces were excluded because a large proportion of men in these areas are traditionally circumcised; Zambia’s tenth province, Muchinga, was created by dividing the Northern province after the study was initiated. The sample was drawn to represent the population in which the Zambian male circumcision program was expected to be scaled up during the course of the study. A two-stage sampling procedure was used, producing a self-weighting sample. First, standard enumeration areas (SEAs) provided by the Central Statistical Office from the 2000 census were randomly selected proportional to size. Because MC services were expected to saturate Central, Copperbelt, Lusaka, and Southern provinces, the sampling frame comprised all SEAs in these provinces. In the remaining three selected provinces—Eastern, Luapula and Northern—the sampling frame was restricted to SEAs within a radius of 50 kilometers from the respective provincial centers (Chipata, Mansa, and Kasama), reflecting the concentration of MC service provision around these towns. Second, a household census was conducted in sampled SEAs to identify eligible study participants, and a constant sampling proportion of randomly selected households with eligible participants was drawn. Respondents were sampled such that two conditions were met: 1) the minimum sample size of 15–24 year-olds—based on power calculations—was determined to assure that a representative sample of the younger cohort was obtained to separately assess this higher risk population; and 2) the proportions of 15–24 and 25–29 year-olds in the sample matched their distribution in the population. For the purposes of this paper, which explores women’s perceptions of MC, we restrict analysis to the female sample.

The first round of data collection was conducted between November 2010 and April 2011; 1,094 eligible women were interviewed and a response rate of 83% of sampled females from the household census was obtained. In the second round, implemented between September and December 2011, 86% of females (946) who were interviewed at baseline were successfully followed. In order to compare trends over time, the analytic sample for this paper consists of 934 females who were interviewed in Round 1 and Round 2 for whom relevant data are complete; 12 women were missing data for one or more of the indicators studied here.

After the Round 1 interview was conducted, 60% of the respondents (women and men) were randomly selected to receive an information package about circumcision and the availability of services in their area. This embedded randomized selective promotion design was employed to account for the fact that men who opt to become circumcised are selective. The randomized promotion provides a potential exogenous or instrumental variable for the unbiased estimation of the impact of circumcision on subsequent sexual behavior. Gender-specific, illustrated information booklets were given to selected respondents and the interviewer briefly explained the material, addressing any questions. The female booklet provided information regarding the benefits and risks of VMMC, including the information that circumcised males are still able to infect their partners with HIV and STIs, the importance of waiting to have sex until after the circumcision wound has healed, and a hotline to call for more information about MC or to find a service.

In addition, at the time of Round 1, mass-media campaigns to increase demand for VMMC were being rolled out. Messages were primarily directed at males, but many of the outlets used—billboards, radio, television, and other promotional efforts—reached the broader population. In addition, the booklets described above were available at selected health facilities and distributed to men being circumcised. The survey instrument included an extensive set of questions on household characteristics, educational attainment, marital status and childbearing, HIV/AIDS knowledge and risk perceptions, gender role attitudes, and sexual behavior. It also collected information about the costs and benefits of male circumcision, knowledge and perceptions of MC, and the circumcision status of respondents or their partners. Non-sensitive data related to background characteristics and MC status were collected via standardized face-to-face interviews and responses entered using computer-assisted personal interviewing (CAPI) with handheld devices. Sensitive questions were asked via audio computer-assisted self-interview (ACASI), whereby respondents listened to questions through headphones and entered responses using a computer touch screen. Statistical analysis of the quantitative data was carried out in Stata version 14 (College Station, TX).

### In-depth interviews

For cost reasons, qualitative respondents were restricted to rural and urban residents of Lusaka and Ndola. Women were selected from three categories of baseline survey respondents: adolescents (females aged 15–17), adult women with a recently circumcised partner, and adult women with an uncircumcised partner. Survey respondents were randomly selected from each of the categories, with the aim of achieving 15 female qualitative respondents per category. Four women were not eligible (partners were circumcised as children), two women were living in another province or country, and three women refused, before achieving a total of 45 females for the sub-sample. All 45 completed their in-depth interviews (IDIs) between February and May of 2011, at least one month after completing the baseline survey. The average length of time between the Round 1 survey and in-depth interview was four months.

IDIs were conducted by trained female interviewers in Bemba, English, or Nyanja, according to the preference of the respondent. A semi-structured interview guide addressed several primary themes: 1) context of the respondent’s intimate relationships; 2) knowledge and perception of MC, including how MC affects female to male and male to female transmission of HIV; 3) sources of information on MC; 4) partner’s MC status and factors in decision to obtain MC or not; 5) condom use; 6) risk perception; and 7) gender norms. The interviewer training emphasized the importance of eliciting detailed responses and pursuing other themes that emerged during the conversation. All interviews were audio-recorded with permission of the respondents, transcribed and translated into English.

Transcripts were read and coded by hand to organize data by categories. The constant comparative method of grounded theory [27, 28] was used to systematically discover emergent themes. Transcripts were also reviewed to identify patterns within the narratives of individual respondents and to compare and elaborate individual participants’ responses to household survey questions [26]. In addition, responses were tabulated to allow comparison of results with the larger quantitative sample and across respondent categories [24, 26].

### Ethics statement

The protocol was reviewed and approved by the Population Council Institutional Review Board (New York), the University of Zambia Biomedical Research Ethics Committee, and the Zambian Ministry of Health. Written informed consent was obtained from each adult respondent. For adolescents, written informed consent of the parent or legal guardian was sought, followed by the written agreement (assent) of the youth.

### Integration and analysis plan

Analysis of the quantitative and qualitative data proceeded in tandem, and was adjusted when patterns or correlations in one data set suggested exploring a question further—or abandoning a line of inquiry—in the other data set. We first assessed four dimensions of women’s knowledge, based on questions from the household survey:

#### Awareness of MC

All respondents who reported having a circumcised partner were characterized as being aware of MC. The remaining women were asked whether they had “heard of male circumcision before it was just described to you.” Only those who answered affirmatively were asked the series of follow-up questions regarding MC’s protective effects.

#### Knowledge that MC partially protects males against HIV

Women demonstrated knowledge that MC is partially protective for males by first responding that MC reduces a man’s risk of getting HIV (as opposed to increasing it or having no effect) and, in a subsequent survey question, disagreeing with the statement: “Male circumcision is fully protective against HIV.”

#### Knowledge that MC reduces males’ STI risk

We identify women who correctly respond that male circumcision reduces a man’s risk of getting sexually transmitted infections other than HIV (as opposed to increasing it or having no effect).

#### Knowledge that MC has no effect on females’ HIV risk if she has sex with a man who is circumcised

We divide women into three groups based on their response to the survey question, “Do you think that male circumcision increases risk, reduces risk, or has no effect on a woman’s risk of getting HIV if she has sex with a man who is circumcised?”

Using McNemar’s test for differences in proportion, and thereby accounting for repeated measures, we compare levels of understanding of each of the four knowledge items across the two survey rounds. At the same time, we present qualitative data on each of the knowledge dimensions to elucidate the quantitative findings. We conclude with an analysis of the correlates of MC awareness and knowledge of MC’s partially protective effect for males, using logistic regression models that include a range of participants’ background characteristics, as well as their partner’s circumcision status, as covariates. We do not estimate logistic regression models for the latter two dimensions of knowledge because as we examined the qualitative data it became clear that the survey questions we used for these two dimensions of knowledge lacked sufficient specificity.

## Results

### Description of sample

Table 1 shows selected sociodemographic characteristics of the quantitative sample at Round 1, according to Round 2 interview status. The 934 women in the analytic sample were on average 21.6 years old, approximately half had married, and 55.7% had attended secondary school or higher. 56.0% of the sample had been tested for HIV and 13.3% currently had a partner who had been circumcised. Relative to their counterparts who were successfully interviewed, the 148 women who were lost to follow up owned fewer household assets, were marginally more likely to belong to the Tonga ethnic group and be born in Southern Province, less likely to be born in Copperbelt province, and marginally more likely to be married.

**Table 1:** Selected sociodemographic characteristics at baseline, by follow-up status and survey sample.

Baseline characteristic	Follow-up status: survey sample		Qualitative sample	Lusaka/Ndola survey sample
	Followed	Lost		
**N**	**934**	**148**^a^	**45**	**262**^b^
Mean age	21.6	21.9	20.8	21.5
Province of birth				
Central	12.7	16.9	4.4	9.2
Copperbelt	33.5	23.0*	28.9	27.5
Lusaka	20.1	17.0	48.9	40.1
Southern	18.2	25.0†	8.9	7.3
Other	15.4	18.2	8.9	16.0
Religion				
Catholic	19.9	14.9	15.6	22.1
Other	80.1	85.1	84.4	77.9
Ethnic group				
Ngoni	21.7	17.6	28.9	35.1
Tonga	24.8	32.4†	20.0	13.7
Bemba	31.4	31.8	37.8	37.4
Other/mixed	22.1	18.2	13.3	13.7
Highest level of school attended				
No school/Primary	39.5	45.3	22.2	36.3†
Secondary	55.7	51.4	60.0	56.5
Trade school/university	4.8	3.4	17.8	7.3*
Mean number of household assets	6.6	5.6**	8.2	7.8
Ever married				
Yes	52.8	60.1†	33.3	50.4*
No	47.2	39.9	66.7	49.6
Ever been tested for HIV				
Yes	56.0	55.9	62.2	47.1†
No	44.0	44.1	37.8	52.9
Partner circumcised				
Yes	13.3	11.5	31.1	15.3**
No/don't know	50.6	52.7	13.3	43.9***
No current partner	36.1	35.8	55.6	40.8†

*** p<0.001;

** p<0.01;

* p<0.05;

^†^ p<0.1

^a^ Compares the baseline characteristics of women interviewed in Round 2 with those who were lost to follow up

^b^ Compares qualitative sample with subset of Round 1 sample resident in Lusaka or Ndola

Figures are percentages unless otherwise indicated; Comparisons use two-sided *z* tests for proportions and *t* tests for means.

Table 1 also shows the characteristics of the 45 women who participated in in-depth interviews after Round 1. These women are a selective group of participants by virtue of the study design: adolescents and women with circumcised partners were oversampled, and we restricted selection to respondents from Lusaka and Ndola, a relatively better educated and wealthier population. Given the sampling restriction, we compare the background characteristics of these women with those of survey respondents residing in Lusaka and Ndola, from which the qualitative subsample was drawn. Compared to the baseline sample of Lusaka and Ndola residents, the qualitative subsample was more likely to have advanced to tertiary education. They were also more likely to have a partner who was circumcised (due to oversampling of women with circumcised partners) and they were less likely to be married (due to oversampling of adolescents).

### Awareness of MC

Awareness of MC is generally high: 77.6% of women at baseline reported having heard of MC before it was described in the interview, rising to 92.0% in Round 2 [Table 2]. Note, however, that increased recognition could be attributable to participation in the baseline interview when MC was described. Moreover, analysis of the three subsequent knowledge indicators revealed considerable uncertainty surrounding the protective effects of MC.

**Table 2:** Knowledge of male circumcision (MC) and its protective benefits among women interviewed in Round 1 and Round 2.

			Round 2 interview	
Knowledge indicator	Round 1 (N = 934)	Round 2 (N = 934)^a^	R1 info pack (N = 620)	No R1 info pack (N = 314)^b^
**Circumcision & HIV (males)**				
1a. Effect of MC on males' HIV risk				
Increases risk	0.5	0.5	0.8	0.0
***Reduces risk***	***64*.*0***	*81*.5***	80.8	82.8
No effect	4.1	2.8	2.9	2.5
Don't know	9.0	7.2	7.7	6.1
Not heard of MC	22.4	8.0 ^c^***	7.7	8.6
1b. MC fully protective against HIV				
Strongly agree/Agree	30.6	40.6***	40.8	40.1
No opinion	10.0	8.6	8.7	8.3
***Strongly disagree/Disagree***	***37*.*0***	*42*.8**	42.7	43.0
Not heard of MC	22.4	8.0 ^c^***	7.7	8.6
1c. ***MC partially protective against HIV (1a & 1b correct)***	***30*.*6***	*37*.5***	37.1	38.2
**Circumcision & STIs (males)**				
2. Male circumcision reduces risk of STIs				
***Strongly agree/Agree***	***61*.*7***	*76*.2***	77.1	74.5
No opinion	10.3	6.7**	8.2	10.5
Strongly disagree/Disagree	5.7	9.0**	6.9	6.4
Not heard of MC	22.4	8.0 ^c^***	7.7	8.6
**Circumcision & HIV (females)**				
3. Effect of MC on females' HIV risk ^d^				
Increases risk	1.1	0.7	0.8	0.6
Reduces risk	50.0	69.8***	70.0	69.4
No effect	12.1	7.9***	7.7	8.2
Don't know	14.4	13.5	13.7	13.1
Not heard of MC	22.4	8.0 ^c^***	7.7	8.6

*** p<0.001;

** p<0.01

^a^ Compares proportions in Round 1 and Round 2 using McNemar’s test

^b^ Compares proportions among R2 respondents who were randomized to receive an MC information pack at after the R1 survey and those who were not; no significant differences were found using two-sided *z* tests

^c^ Note that increased awareness of MC may stem from participation in the Round 1 interview, when the subject of MC was raised

^d^ Due to a lack of precision in the survey questions, multiple interpretations are possible

Correct responses are shown in ***italics***; Figures are percentages.

### Perception of the impact of MC on HIV for men

There was a significant increase across rounds in the proportion of female survey respondents who correctly answered basic questions about MC’s HIV prevention benefits for men [Table 2]. Eight out of 10 women (81.5%) in Round 2 correctly said that circumcision reduces men’s HIV risk, up significantly from 64.0% the previous round (p<0.001). However, the proportion of women able to correctly answer a more nuanced question—whether MC is fully protective against HIV—was much smaller. Only 37.0% of women in Round 1 and 42.8% in Round 2 correctly responded that it was not true that MC is fully protective against HIV for men (p<0.01). A large proportion of women (30.6%) in Round 1 and significantly more (40.6%, p<0.001) in Round 2 incorrectly believed that MC is fully protective. Approximately one-third of women accurately indicated that MC partially protects males against HIV by correctly answering both survey questions about men’s HIV risk (30.6% in Round 1, and 37.5% in Round 2, p<0.001). No significant differences were observed at Round 2 between the respondents randomized to the information packet compared to those who were not.

Comparing individual women’s responses in the quantitative survey to their response in the in-depth interviews suggests even more confusion than is apparent in the quantitative findings. Even among women in the subsample who correctly indicated at baseline that MC is partially protective against HIV for men, almost half (11 out of 24) nonetheless expressed uncertainty or an erroneous understanding in their IDIs—including that MC fully protects men from HIV or that MC offers no protection.

Examining data from all 45 women in the qualitative subsample reveals the same pattern. Overall, about half of the women (26 out of 45) understood that MC is partially protective against HIV. Among IDI participants who did not understand the partial efficacy of MC in preventing HIV among men in their in-depth interviews (19), more than half (11) believed that MC protects men completely from HIV infection. As one woman (age 18, partner is circumcised, no R1 information packet) explained, “Because circumcision protects from HIV, if a man is circumcised he cannot get HIV even if he doesn’t use a condom during sex.”

Some (5) women, by contrast, did not believe that MC protects men from HIV at all: “Circumcision does not protect anyone from HIV in any way; the two are not linked in any way” (Female, age 16, R1 information packet). Three other respondents interpreted "partial protection" to mean that a man who is circumcised cannot get HIV from an HIV-positive woman if he has sex with her only once.

Circumcision reduces the chances of contracting HIV, if a man who is circumcised has sex with an HIV positive person just once he cannot get HIV, he can only get it if he has sex with her two, three times.

Age 25, partner is circumcised, R1 information packet

### Perception of the impact of MC on STIs for men

Most female survey respondents correctly replied that circumcision reduces men’s risk of STIs other than HIV [Table 2]. The proportion who responded correctly increased significantly from Round 1 to Round 2 (from 61.7% to 76.2%, p<0.001). Yet the IDIs suggest once again that this question masks considerable misunderstanding. Examining the qualitative responses of those women who reported in the survey that MC reduces men’s risk of STIs (34), about a third (12) correctly explained that MC partially protects men from STIs. Among the remainder, one woman indicated that MC offers no protection against STIs, while the majority (21) incorrectly believed that MC reduces men’s risk of acquiring STIs completely or almost completely.

Interviewer: If a man who has been circumcised has sex with a woman who has an STI such as syphilis or gonorrhea, can he get that STI from her?

Respondent: No he can’t get it.

Interviewer: Why?

Respondent: He can’t get the STI because that skin where the STI can enter from and sort of settle is not there, the penis is open without any entry point for the STI.

Age 30, partner is not circumcised, R1 information packet Looking across all 45 in-depth interviews, the same pattern holds.

### Perception of the impact of MC on HIV for women

In both survey rounds, most women said that MC reduces a woman's HIV risk if she has sex with a man who is circumcised (50.0% in Round 1, increasing significantly to 69.8% in Round 2, p<0.001) [Table 2]. At baseline, 12.1% of women reported that MC has no effect on a female’s HIV risk in such circumstances. This proportion declined significantly to 7.9% (p<0.001) in Round 2. The proportion of women responding “don’t know” was also relatively high: 14.4% and 13.5% in Rounds 1 and 2, respectively.

Given the lack of precision of our survey question, we cannot discern from the quantitative data whether women incorrectly attribute a *direct* reduction in their individual HIV risk to MC, or whether women perceive their risk to be reduced *indirectly* with a circumcised partner because of the decreased likelihood that he is HIV-positive or that he will acquire HIV, a relatively complex yet possible reasoning pattern. The IDIs help elucidate women’s responses to the survey.

Twenty-nine of the forty five women in the qualitative subsample had replied in the survey that MC reduces women’s risk of HIV. Several (4) of these women correctly noted that women are not directly protected by MC against HIV and attributed a reduction in women’s risk of HIV to indirect protection:

Interviewer: How would you describe your risk of becoming HIV infected, would you say you are at high risk, moderate risk or low risk?

Respondent: Moderate risk.

Interviewer: Please explain why you think so?

Respondent: Since he is circumcised I worry less about HIV, you can’t trust a man completely, but at least he is circumcised so even if he has a girlfriend that I don’t know about, I know that he is protected.

Age 20, partner is circumcised, no R1 information packet

However, ten of the women who had reported in the survey that MC reduces women’s HIV risk incorrectly attributed that reduction to direct protection at the individual level. Most of these women believed that MC offers women partial direct protection. For example, one participant explained that if a man is circumcised, his partner also has 65% protection against HIV because “…that skin in front keeps dirt and easily gets cuts, but when it is removed one cannot easily get cuts and transmit the virus so that way the woman also benefits” (age 22, partner is circumcised, R1 information packet). Several others thought that MC completely protects women from HIV. One respondent (age 25 years, partner is circumcised, no R1 information packet) explained, “She can’t get HIV from a circumcised man […], so it [MC] protects her fully.”

Finally, about a third (10) of the women who had replied that MC reduces women’s risk in the survey explained in their IDIs that MC does not offer women any protection, for example because “the circumcision is not done on the woman’s body so it does not protect her and she is at full risk of contracting the virus” (Age 18, partner is not circumcised, R1 information packet).

Of the four women in the qualitative subsample who had replied in the survey that MC has no effect on a woman’s HIV risk, only one expressed this belief in their IDI. The others indicated either that MC provides women with partial protection (2), or was unclear in the interview.

### What is associated with women's perceptions of MC?

Having observed that women’s understanding of the protective effects of MC on men and women is far from complete, we now investigate the determinants of knowledge using our quantitative data. Table 3 shows unadjusted and adjusted odds ratios from logistic regression models for the following outcomes at Round 1:

1. Awareness of MC
2. Knowledge that MC partially protects males against HIV

**Table 3:** Odds ratios from unadjusted and adjusted logistic regression models identifying associations between sociodemographic characteristics and 1) awareness of male circumcision (MC) and 2) knowledge that MC partially protects males against HIV.

	Outcome 1 (N = 934)		Outcome 2 (N = 934)	
Sociodemographic characteristics	Awareness of MC		Knowledge that MC partially protects males against HIV	
	Unadjusted	Adjusted	Unadjusted	Adjusted
Age group				
15–24	1.00	1.00	1.00	1.00
25–29	1.56*	1.59*	1.40*	1.54*
Province of birth				
Central	1.00	1.00	1.00	1.00
Copperbelt	1.90**	1.44	2.67***	1.99*
Lusaka	2.80**	2.37**	1.55	1.26
Southern	1.30	1.52	1.84*	1.85†
Other	1.36	1.14	1.50	1.33
Religion				
Catholic	1.00	1.00	1.00	1.00
Other	0.87	0.94	1.06	1.07
Ethnic group				
Ngoni	1.00	1.00	1.00	1.00
Tonga	0.60*	0.65	1.19	1.16
Bemba	0.76	0.83	1.40	1.21
Other	0.99	1.05	1.90**	1.62*
Highest level of school attended				
Primary or less	1.00	1.00	1.00	1.00
Secondary	1.92***	1.70**	2.53***	1.99***
Trade school/university	6.02**	3.50†	11.10***	5.31***
Number of household assets	1.13***	1.09**	1.18***	1.13***
Ever married				
No	1.00	1.00	1.00	1.00
Yes	1.06	0.73	0.89	0.97
Current relationship				
No	1.00	1.00	— ^a^	— ^a^
Yes	1.71**	2.24***	—	—
Ever been tested for HIV				
No	1.00	1.00	1.00	1.00
Yes	1.60**	1.38†	1.72***	1.52*
Circumcision status of partner	N/A	N/A		
No/don't know			1.00	1.00
Yes			2.20***	2.01**
No current relationship			1.14	1.06
LR chi2		82.1***		138.1***
Pseudo R2		0.08		0.12

*** p<0.001;

** p<0.01;

* p<0.05;

† p<0.1

^a^ Variable omitted due to duplication with partner’s circumcision status.

We find, perhaps not surprisingly, that in both unadjusted and adjusted models women who were older, more highly educated, owned a higher number of household assets, and were currently in a relationship, were significantly more likely to have heard of MC than their younger, less educated, poorer, and single counterparts. At the time of the Round 1 survey, respondents from Lusaka and Copperbelt were also more likely to have heard of MC relative to women in Central province, but only Lusaka residence remained significant in the adjusted model.

With respect to knowledge that MC provides partial protection against HIV for males, age, a higher level of education, higher number of household assets, having been tested for HIV, and having a circumcised partner, were associated with correct understanding in unadjusted models. Being from Copperbelt or Southern (relative to Central) province, and “other” ethnicity (relative to Ngoni), were also significant. In the adjusted model, age, higher education, higher number of household assets, HIV testing, being born in Copperbelt, belonging to “other” ethnicity, and having a circumcised partner remained significant.

## Discussion

Women potentially contribute substantially to MC uptake by playing an active role in their partner’s decision to obtain MC, assenting when their partner indicates an interest in circumcision, or participating in the decision about circumcising a son. They may also act as supportive partners in seeking MC services or in the post-operative period. Finally, women’s accurate knowledge of the protection MC does and does not afford is important to avoid risk compensation.

With respect to basic MC awareness and knowledge, some of our findings are encouraging. At baseline, during the very early periods of the VMMC program in Zambia, three-quarters of women had heard about MC and 64.0% of women correctly indicated that MC reduces males’ risk of HIV, increasing to 81.5% in Round 2. Similar proportions accurately responded that MC provides protection against some STIs.

However, women demonstrated a lack of more nuanced knowledge of MC’s protective effects, which is cause for concern. A strikingly large proportion of women in the quantitative survey incorrectly believed that MC fully protects men from HIV. The fact that this proportion significantly increased over time is particularly troubling. Moreover, this misperception is mirrored in the qualitative subsample, with close to half of the women not understanding that MC provides only partial protection for men. Even among those women in the subsample who had responded *correctly* in the quantitative survey, almost half expressed uncertainty or significant misperceptions about MC’s protective effect for men against HIV in their in-depth interviews.

We observe a similar pattern regarding women’s perception of the impact of MC on STI risk for men. Although the majority of survey respondents correctly replied that MC reduces men’s risk of some STIs, the qualitative data suggest that considerable misconceptions remain. Indeed, two-thirds of women in the qualitative subsample had misperceptions about the reduction in STI risk among men that is attributable to MC. The vast majority of these women mistakenly believed that MC completely, or almost completely, protects men against STIs. Thus, our findings indicate that even in cases where superficial knowledge is demonstrated, substantial confusion exists over the relationship between MC and men’s HIV/STI acquisition, and the degree of protection afforded. This is true even among our relatively more educated and wealthier qualitative subsample.

Quantitative data related to women’s perceptions of the impact of MC for women’s HIV risk are not easily interpreted due to the inability of the survey question to distinguish direct from indirect risk transmission. The qualitative data suggest both that: 1) some women (correctly) interpret a female’s risk of HIV being reduced because of the lower likelihood that her partner would become infected if he were HIV-negative and obtained MC; but also that 2) other women incorrectly believe that MC offers women partial—or even complete—direct protection against HIV. The pervasiveness of the latter pattern is of concern, as this belief could lead to risk compensation or reduce women’s ability or motivation to negotiate condom use. Comparing MC knowledge among women who were randomized to receive the MC info packs in R1 and those who were not, no significant differences were found for any knowledge indicator.

Multivariable analysis shows that, in general, women who are more highly educated and wealthier are better informed about MC than their more disadvantaged peers. The association between partner’s circumcision status and knowledge of MC’s partial protection against HIV for males is encouraging.

## Limitations

This study has three main limitations. First, the survey questions related to MC’s effects on men’s risk of STI acquisition and on women’s HIV risk were not sufficiently nuanced, with the qualitative data revealing multiple possible interpretations. Second, the fact that the qualitative subsample was drawn from Lusaka and Ndola respondents, and not all seven provinces in which the survey was fielded, means that the qualitative responses do not reflect the entire survey sample. However, because the subsample respondents are relatively more privileged in terms of education and socioeconomic status (both of which are associated with greater knowledge) we are likely to have underestimated, rather than overestimated, the misunderstandings that prevail. Finally, classifications of partners’ circumcision status were based on women’s self-reports, which have been shown to be subject to error.[29, 30] However, in-depth interviews revealed that all women in the qualitative subsample accurately categorized themselves, suggesting that misclassification had a minimal, if any, effect on our analysis.

## Recommendations

Echoing the suggestions of Maughan-Brown and Venkataramani [22], research should employ more nuanced questions to better capture knowledge of MC. In particular, greater specification regarding women’s risk of HIV if her partner is circumcised is needed. Similarly, a question regarding the degree of risk for STI transmission—whether MC is partially or fully protective—would be illuminating.

Our findings suggest that more efforts are needed to improve women’s understanding about the limits of MC protection, particularly among women who are younger, less educated and from lower SES households. Given that these characteristics also tend to be associated with higher risk of HIV acquisition, this recommendation is arguably critical for VMMC programs. While all women have a right to full and accurate information about MC, efforts to improve women’s understanding of MC should be sure to reach those more marginalized women who may have difficulty accessing and processing accurate information about MC and harbor misperceptions about its protective effects.

Finally, messaging surrounding MC should more explicitly address women’s needs. Specifically, health professionals should emphasize that, because MC provides men with only partial protection against HIV and some STIs, women are still at risk if a partner is HIV positive regardless of his circumcision status. The concept of partial protection is difficult to communicate and research is needed to test the effectiveness of different approaches. The fact that no knowledge differences were observed when comparing women who received information packets with women who did not, speaks to the challenge of conveying this information. Research is needed to identify effective ways to do so. The message that condoms are critical for prevention remains important. Tailored instructional or promotional materials about MC targeted directly at women should be produced and distributed in multiple fora to reach women to convey these messages. One opportunity to provide women with information is to utilize “female” spaces—e.g. in antenatal clinics, maternity wards, and postnatal and under-five clinics. The need to respond to women’s desire for more and accurate information on MC is urgent, and ethically indicated.

## Supporting Information

S1 Dataset De-identified data for “Women’s Perceptions and Misperceptions of Male Circumcision”. (DTA)

## References

[1] Auvert B, Taljaard D, Lagarde E, Sobngwi-Tambekou J, Sitta R, Puren A. Randomized, Controlled Intervention Trial of Male Circumcision for Reduction of HIV Infection Risk: The ANRS 1265 Trial. PLoS Medicine. 2005;2(11):1112–22. 10.1371/journal.pmed.0020298 PMC1262556 16231970

[2] Bailey RC, Moses S, Parker CB, Agot K, Maclean I, Krieger JN, et al Male Circumcision for HIV Prevention in Young Men in Kisumu, Kenya: A Randomised Controlled Trial. Lancet. 2007;369:643–56. 17321310 10.1016/S0140-6736(07)60312-2

[3] Gray RH, Kigozi G, Serwadda D, Makumbi F, Watya S, Nalugoda F, et al Male Circumcision for HIV Prevention in Men in Rakai, Uganda: A Randomised Trial. Lancet. 2007;369(657–666). 17321311 10.1016/S0140-6736(07)60313-4

[4] World Health Organization, UNAIDS. New Data on Male Circumcision and HIV Prevention: Policy and Programme Implications: Conclusions and Recommendations. WHO/UNAIDS Technical Consultation Male Circumcision and HIV Prevention: Research Implications for Policy and Programming 6–8 March; Montreux2007.

[5] Ministry of Health (MOH) Zambia. National Male Circumcision Strategy and Implementation Plan 2010–2020. Ministry of Health (MOH), Zambia, 2010.

[6] Ministry of Health (MOH) Zambia. Country Operational Plan for the Scale-up of Voluntary Medical Male Circumcision in Zambia, 2012–2015. Ministry of Health (MOH), Zambia, 2012.

[7] Wawer MJ, Makumbi F, Kigozi G, Serwadda D, Watya S, Nalugoda F, et al Circumcision in HIV-Infected Men and Its Effect on HIV Transmission to Female Partners in Rakai, Uganda: A Randomised Controlled Trial. Lancet. 2009;374:229–37. 10.1016/S0140-6736(09)60998-3 19616720 PMC2905212

[8] Weiss HA, Hankins CA, Dickson K. Male Circumcision and Risk of HIV Infection in Women: A Systematic Review and Meta-Analysis. Lancet Infectious Diseases. 2009;9(11):669–77. 10.1016/S1473-3099(09)70235-X 19850225

[9] Baeten JM, Donnell D, Kapiga SH, Ronald A, John-Stewart G, Inambao M, et al Male Circumcision and Risk of Male-to-Female HIV-1 Transmission: A Multinational Prospective Study in African HIV-1-Serodiscordant Couples. AIDS. 2010;24(5):737–44. 20042848 10.1097/QAD.0b013e32833616e0 PMC2919808

[10] Hallett TB, Alsallaq RA, Baeten JM, Weiss H, Celum C, Gray R, et al Will Circumcision Provide Even More Protection From HIV to Women and Men? New Estimates of the Population Impact of Circumcision Interventions. Sexually Transmitted Infections. 2011;87:88–93. 10.1136/sti.2010.043372 20966458 PMC3272710

[11] Gray RH, Kigozi G, Serwadda D, Makumbi F, Nalugoda F, Watya S, et al The Effects of Male Circumcision on Female Partner's Genital Tract Symptoms and Vaginal Infections in Randomized Trial in Rakai, Uganda. American Journal of Obstetrics and Gynecology. 2009;200(1):42.e1–.e7. 10.1016/j.ajog.2008.07.069 18976733 PMC2727852

[12] Tobian AAR, Serwadda D, Quinn TC, Kigozi G, Gravitt PE, Laeyendecker O, et al Male Circumcision for the Prevention of HSV-2 and HPV Infections and Syphilis. The New England Journal of Medicine. 2009;360(13):1298–309. 10.1056/NEJMoa0802556 19321868 PMC2676895

[13] Wawer MJ, Tobian AAR, Kigozi G, Kong X, Gravitt PE, Serwadda D, et al Effects of Circumcision of HIV-Negative Men on Transmission of Human Papillomavirus to HIV-Negative Women: A Randomised Trial in Rakai, Uganda. Lancet. 2011;377:209–18. 10.1016/S0140-6736(10)61967-8 21216000 PMC3119044

[14] Sobngwi-Tambekou J, Taljaard D, Nieuwoudt M, Lissouba P, Puren A, Auvert B. Male Circumcision and Neisseria Gonorrhoeae, Chlamydia Trachomatis and Trichomonas Vaginalis: Observations After a Randomised Controlled Trial for HIV Prevention. Sexually Transmitted Infections. 2009;85:116–20. 10.1136/sti.2008.032334 19074928 PMC2652030

[15] Castellsague X, Bosch FX, Munoz N, Meijer CJLM, Shah KV, De Sanjose S, et al Male Circumcision, Penile Human Papillomavirus Infection, and Cervical Cancer in Female Partners. The New England Journal of Medicine. 2002;346(15):1105–12. 11948269 10.1056/NEJMoa011688

[16] Gray R, Kigozi G, Kong X, Ssempijja V, Makumbi F, Wattya S, et al The Effectiveness of Male Circumcision for HIV Prevention and Effects on Risk Behaviors in a Posttrial Follow-up Study. AIDS. 2012;26(5):609–15. 10.1097/QAD.0b013e3283504a3f 22210632 PMC4296667

[17] Mattson CL, Campbell RT, Bailey RC, Agot K, Ndinya-Achola JO, Moses S. Risk Compensation is Not Associated with Male Circumcision in Kisumu, Kenya: A Multi-Faceted Assessment of Men Enrolled in a Randomized Controlled Trial. PLoS ONE. 2008;3(6):e2443 10.1371/journal.pone.0002443 18560581 PMC2409966

[18] Westercamp N, Agot K, Jaoko W, Bailey RC. Risk Compensation Following Male Circumcision: Results from a Two-Year Prospective Cohort Study of Recently Circumcised and Uncircumcised Men in Nyanza Province, Kenya. AIDS Behavior. 2014;18(9):1764–75. 10.1007/s10461-014-0846-4 25047688

[19] Westercamp M, Agot KE, Ndinya-Achola J, Bailey RC. Circumcision Preference Among Women and Uncircumcised Men Prior to Scale-Up of Male Circumcision for HIV Prevention in Kisumu, Kenya. AIDS Care. 2012;24(2):157–66. 10.1080/09540121.2011.597944 21854351 PMC3682798

[20] Young MR, Odoyo-June E, Nordstrom SK, Irwin TE, Ongong'a DO, Ochomo B, et al Factors Associated with Uptake of Infant Male Circumcision for HIV Prevention in Western Kenya. Pediatrics. 2012;130(1):e175–e82. 10.1542/peds.2011-2290 22711723

[21] Andersson N, Cockcroft A. Male Circumcision, Attitudes to HIV Prevention and HIV Status: A Cross-Sectional Study in Botswana, Namibia, and Swaziland. AIDS Care. 2012;24(3–4):301–9. 21933035 10.1080/09540121.2011.608793 PMC3379742

[22] Maughan-Brown B, Venkataramani AS. Learning that Circumcision is Protective against HIV: Risk Compensation among Men and Women in Cape Town, South Africa. PLoS ONE. 2012;7(7):e40753 10.1371/journal.pone.0040753 22829883 PMC3400649

[23] Okeyo T, Westercamp N, Bailey RC, Agot K, Jaoko W. What Women Think About Male Circumcision: Perceptions of the Female Partners of Recently Circumcised Men in Nyanza Province, Kenya. International AIDS Conference 2012; July 22–27, 2012; Washington, D.C.2012.

[24] Creswell JW, Plano Clark V. Designing and Conducting Mixed Methods Research. Second ed Thousand Oaks, CA: Sage Publications; 2011.

[25] Tashakkori A, Teddlie C. Handbook of Mixed Methods in Social and Behavioral Research. Thousand Oaks, CA: Sage Publications; 2003.

[26] Schatz E. Rationale and Procedures for Nesting Semi-Structured Interviews in Surveys or Censuses. Population Studies: A Journal of Demography. 2012 10.1080/00324728.2012.658851 PMC3750727 22364562

[27] Glaser BG, Strauss AL. The Discovery of Grounded Theory: Strategies for qualitative research. New Brunswick, NJ: Transaction Publishers; 1967.

[28] Glaser BG. Basics of Grounded Theory Analysis. Mill Valley, CA: Sociology Press; 1992.

[29] Hewett PC, Haberland N, Apicella L, Mensch BS. The (Mis)Reporting of Male Circumcision Status Among Men and Women in Zambia and Swaziland: A Randomized Evaluation of Interview Methods. PLoS ONE. 2012;7(5):e36251 10.1371/journal.pone.0036251 22629312 PMC3358314

[30] Kong X, Ndyanabo A, Nalugoda F, Kigozi G, Ssekasanvu J, Lutalo T, et al The accuracy of women’s reports of their partner’s male circumcision status in Rakai, Uganda. AIDS. 2013;27(4):662–4. 10.1097/QAD.0b013e32835c557c PubMed Central PMCID: PMCPMC3932992. 23169325 PMC3932992

